# Calculating the Malliavin derivative of some stochastic mechanics problems

**DOI:** 10.1371/journal.pone.0189994

**Published:** 2017-12-20

**Authors:** Paul Hauseux, Jack S. Hale, Stéphane P. A. Bordas

**Affiliations:** 1 Institute of Computational Engineering, University of Luxembourg, 6 Avenue de la Fonte, 4362 Esch-sur-Alzette, Luxembourg; 2 Cardiff School of Engineering, Cardiff University, The Queen’s Building, The Parade, Cardiff, Wales, CF24 3AA, United Kingdom; China University of Mining and Technology, CHINA

## Abstract

The Malliavin calculus is an extension of the classical calculus of variations from deterministic functions to stochastic processes. In this paper we aim to show in a practical and didactic way how to calculate the Malliavin derivative, the derivative of the expectation of a quantity of interest of a model with respect to its underlying stochastic parameters, for four problems found in mechanics. The non-intrusive approach uses the Malliavin Weight Sampling (MWS) method in conjunction with a standard Monte Carlo method. The models are expressed as ODEs or PDEs and discretised using the finite difference or finite element methods. Specifically, we consider stochastic extensions of; a 1D Kelvin-Voigt viscoelastic model discretised with finite differences, a 1D linear elastic bar, a hyperelastic bar undergoing buckling, and incompressible Navier-Stokes flow around a cylinder, all discretised with finite elements. A further contribution of this paper is an extension of the MWS method to the more difficult case of non-Gaussian random variables and the calculation of second-order derivatives. We provide open-source code for the numerical examples in this paper.

## Introduction

The classical derivative is a fundamental tool of calculus that is widely used across every field of mathematics, science and engineering. Various generalisations and extensions of the classical derivative, e.g. local and/or partial Frechét and Gâteaux derivatives [1], are now common tools in the repertoire of practitioners working in many fields. Modern extensions such as fractional and non-local derivatives are finding increasing use in several fields of science and technology, see e.g. [2–6]. The semi-inverse method of [7] is a powerful tool for the establishment of variational principles (Euler-Lagrange) from governing equations for physical problems.

By contrast, the Malliavin calculus [8], an extension of the notions of classical calculus of variations to stochastic processes, is certainly less widely known. In our view, this is probably because the vast majority of papers written on the subject require study of mathematics and stochastics to an advanced level. However, we think that Malliavin calculus deserves a wider audience. The objective of this paper then is introduce the Malliavin derivative as a useful numerical tool for practitioners to understand the behaviour of stochastic PDEs in mechanics, rather than to fully explain the technicalities of Malliavin calculus. Interested readers are referred to e.g. [8–10] for a full mathematical treatment.

We are not the first to apply Malliavin calculus as a useful tool for practical computation. The Malliavin calculus can be used to efficiently calculate the Greeks, the sensitivity of financial instruments to their underlying parameters e.g. [11–14]. In the physical sciences we are aware of only a handful of recent papers that use techniques inspired by the Malliavin calculus to understand the behaviour of systems with stochastic behaviour. We are not aware of any papers in the engineering mechanics community on the topic. [15] introduced the methodology of Malliavin Weight Sampling (MWS), the method we adopt in this paper, and applied it to the simulation of particles undergoing Brownian motion. [16] presented a more general framework for deriving the MWS update rules and its practical implementation. [17] used the MWS to evaluated linear response functions of particle systems forced by coloured noise. When the coefficients of the models are assumed to follow known statistical distributions, then the likelihood ratio method can be seen as a Malliavin weighting function [11, 12, 18]. The Malliavin theory is however more general and allows the determination of the optimal weight with minimum variance even if the specification of the stochastic parameters involved in the model are not known explicitly.

The contribution of this paper is as follows; we show the application of the Malliavin Weight Sampling method [15] to four archetypal problems in mechanics. Unlike the examples in [15], we consider some models defined by partial differential equations (PDEs) that are discretised using the finite element method. We make a new extension of the MWS method to parameters defined by non-Gaussian distributions. This has important practical value because it is often important to model parameters with distributions that preclude realisations with non-physical values, e.g. positive viscosity in a fluid mechanics problem. Finally we extend the MWS method in [15] to the calculation of second-order derivatives.

An outline of this paper is as follows; we give an outline of the MWS method and use the MWS method to study the behaviour of a simple Kelvin-Voigt visco-elastic system with Gaussian and non-Gaussian stochastic variables respectively. We extend the analysis of the Kelvin-Voigt system to the second derivative. We then study; a 1D elastic bar, a hyperelastic bar prone to buckling, and Navier-Stokes flow around a cylinder, all discretised in space using the finite element method. Finally we summarise and suggest some interesting avenues for future research.

## The Malliavin Weight Sampling (MWS) method

### Problem setting

Consider a non-linear, possibly time-dependent stochastic partial differential equation *F*(*u*, *m*) = 0 with random parameter *m*. For each possible value of *m*, *u* is the solution of the PDE and therefore *u* depends explicitly on *m* (*m* ↦ *u*(*m*)). To simplify the notation, the spatial position *x* and time *t* are omitted but it is understood that *u* can also depend on $x\in \Omega \subset R^{d}$ where *d* = {1, 2, 3} is the spatial dimension of the domain and/or $t\in R^{+}$.

Let $\left(\Omega_{p},F,P\right)$ a probability space where Ω_*p*_ is the sample space, F is a *σ*-algebra of subsets of Ω_*p*_ and *P* is a probability measure. We are interested to evaluate the expected value of a quantity of interest *J*(*m*) = *J*(*u*(*m*)) denoted by E[J] [19]:

$$\begin{array}{c} E\left[J\right]≔\int_{\Omega_{p}}J\left(u\left(\omega \right)\right)\cdot dP\left(\omega \right). \end{array}$$ (1)

In a practical way, if *m* is a random variable with probability density function *f*_*m*_, Eq (1) writes:

$$\begin{array}{c} E\left[J\right]≔\int_{R}J\left(u\left(x\right)\right)\cdot f_{m}\left(x\right)dx. \end{array}$$ (2)

As we will see, the Malliavin Weight Sampling method (MWS) [16] allows the evaluation of the sensitivity of the expected value of the quantity of interest with respect to the mean value of the stochastic parameter *m* as [9, 11, 16, 18]:

$$\begin{array}{c} \frac{\partial E[J]}{\partial \bar{m}}=E\left[Jq_{m}\right], \end{array}$$ (3)

where *q*_*m*_ is the Malliavin weight for the parameter *m* and $\bar{m}$ is the mean of *m*. Under certain condition of regularity [11, 20, 21] when the probability density function (PDF) of the parameter *m* is known, the Malliavin weight *q*_*m*_ associated can be computed directly from the PDF of *m*. This approach can be viewed as an integration by parts, and is a direct result of Malliavin calculus where we take the derivative of random functions rather than the classical derivative. We emphasise again the quite different nature of the above derivative Eq (3) to the classical notion of a derivative from elementary calculus.

In Eq (3), we suppose that the quantity of interest *J* does not depend explicitly on the parameter *m*. Later we introduce a more general equation Eq (35) that must be considered if in fact *J* does depend on *m*.

The simplest approach to calculate $E[Jq_{m}]$, and the one we use exclusively in this paper, is to use the standard Monte Carlo estimator; that is, take *Z* independent and identically distributed (iid) realisations *m*_*z*_ of *m*, solve for *J*_*z*_≔ *J*(*u*(*m*_*z*_)) before taking the sample mean of the set of realisations {*J*_1_, …, *J*_*Z*_}:

$$\begin{array}{c} \frac{\partial E[J]}{\partial \bar{m}}=E\left[Jq_{m}\right]\approx \frac{1}{Z}\sum_{z=1}^{Z}J\left(m_{z}\right)\cdot q_{m}\left(m_{z}\right). \end{array}$$ (4)

From the central limit theorem, the error in Eq (4) is normally distributed with variance *Z*^−1^ *V* where V is the variance of *Jq*_*m*_.

What will not be clear to the reader at this stage is how to determine the Malliavin weights. Through a simple practical examples in the next section, we will explain how to use the MWS method, determine the specification of the weights for both Gaussian and non-Gaussian distributions on the parameter *m*, and thus calculate the Malliavin derivative Eq (3).

## Kelvin-Voigt model

The Kelvin-Voigt constitutive model with Young’s modulus *E*, viscosity *η* and loading stress *σ* can be written as the following linear ordinary differential equation:

$$\begin{array}{c} E\epsilon \left(t\right)+\eta \frac{d\epsilon (t)}{\text{dt}}=\sigma . \end{array}$$ (5)

**Fig 1:**
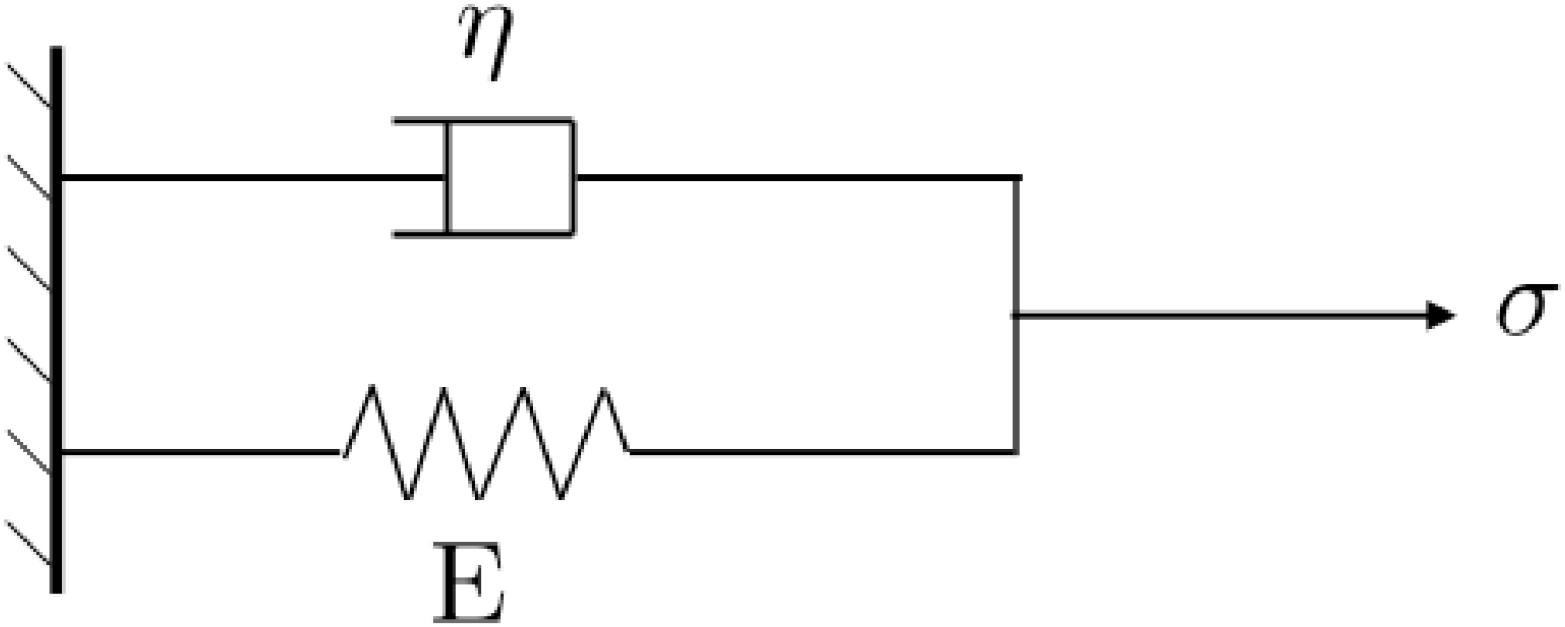
Schematic of a Kelvin-Voigt model with Young’s modulus *E*, viscosity *η* and loading stress *σ*. We model the loading stress *σ* as a random noise (random variable), inducing a random strain *ϵ* as the output of the model.

A schematic of this model is shown in Fig 1.

The initial condition on the strain is *ϵ*(*t* = 0) = 0 and we study the response of the system for time *t* ∈ [0, *T*]. Our quantity of interest functional is the value of the strain at time *t*, i.e.:

$$\begin{array}{c} J≔\epsilon (t) \end{array}$$ (6)

and we are interested in its expected value (mean) E[ϵ(t)].

### Gaussian case

We first consider the case that the randomness can be modelled as a Gaussian random variable. A similar model is shown in [16].

We choose choose to model the stress as a random noise:

$$\begin{array}{c} \sigma \left(t\right)=\sigma_{0}+\alpha \xi , \end{array}$$ (7)

where *σ*_0_ and *α* are constant and *ξ* is a a Gaussian random variable with zero mean and unit variance. *ξ* represents the uncertainty related to the value of the stress *σ*.

From Eq (7), the mean value of *σ* is *σ*_0_ and the variance of *σ* is equal to *α*^2^. We assume throughout that the Young’s modulus *E* and the viscosity *η* are perfectly known. Given that the forcing stress *σ* for the system is random, the strain *ϵ* is also random. The goal then is to evaluate the derivative of the expected value of the strain with respect to the mean value *σ*_0_:

$$\begin{array}{c} \frac{\partial E[\epsilon (t)]}{\partial \sigma_{0}} \end{array}$$ (8)

using the method of MWS.

We choose to solve the ODE Eq (5) using an Euler explicit finite difference method with time step *δt*:

$$\begin{array}{c} \epsilon \left(t+\delta t\right)=\epsilon \left(t\right)+\frac{\delta t}{\eta }\left[\sigma_{0}-E\epsilon \left(t\right)+\frac{\alpha \xi }{\sqrt{\delta t}}\right]. \end{array}$$ (9)

*Remark*. Note that the multiplying term before *ξ* contains $\sqrt{\delta t}$ and not *δt*. This is a ‘conforming’ discretisation of the stochastic noise term, resulting in a dependence of the variance of the random parameter on the discretisation size. Informally, taking the limit, we can recover the original ODE Eq (5) as:

$$\begin{array}{c} E\left[\frac{d\epsilon }{\text{dt}}\right]=\left(\sigma_{0}-E\epsilon \left(t\right)\right)/\eta , \end{array}$$ (10)

and:

$$\begin{array}{c} V\left[\eta \frac{d\epsilon }{\text{dt}}-\left(\sigma_{0}-E\epsilon \left(t\right)\right)\right]=\alpha^{2}. \end{array}$$ (11)

Where V[·] is the the variance. Given that $E[\sqrt{\delta t}\xi ]=0$ and $E\left[{\left(\sqrt{\delta t}\alpha \xi \right)}^{2}\right]=\alpha^{2}\delta t$, the numerical method in Eq (9) is consistent in the following sense:

$$\begin{array}{c} \lim_{\delta t\to 0}\frac{1}{\delta t}E\left[\epsilon \left(t+\delta t\right)-\epsilon \left(t\right)\right]=\left(\sigma_{0}-E\epsilon \left(t\right)\right)/\eta , \end{array}$$ (12)

$$\begin{array}{c} \lim_{\delta t\to 0}\frac{1}{\delta t}E\left[{\left[\eta \left(\epsilon \left(t+\delta t\right)-\epsilon \left(t\right)\right)-\delta t\left(\sigma_{0}-E\epsilon \left(t\right)\right)\right]}^{2}\right]=\alpha^{2}. \end{array}$$ (13)

For this next part, we adopt the same notation as [16]. We denote *ϵ* the strain of the the system at time *t* and we denote *ϵ*′ the strain of the the system at time *t* + *δt*. Furthermore, we let *P*(*ϵ*) and *P*(*ϵ*′) be the probability that the strain of the system is *ϵ* and *ϵ*′ respectively. The propagator *W*(*ϵ* → *ϵ*′) must satisfy:

$$\begin{array}{c} P\left(\epsilon^{\prime }\right)=\int_{\epsilon }W\left(\epsilon \to \epsilon^{\prime }\right)P\left(\epsilon \right)d\epsilon , \end{array}$$ (14)

$$\begin{array}{c} \int_{\epsilon^{\prime }}W\left(\epsilon \to \epsilon^{\prime }\right)d\epsilon^{\prime }=1. \end{array}$$ (15)

Eq (14) means that the probability that the strain of the system is *ϵ*′ is the sum (integral) of all the probabilities to be at *ϵ* multiplied by the probability that the system passes from state *ϵ* to *ϵ*′ during *δt*. Condition Eq (15) comes from the integration of the first condition over *ϵ*′.

To derive the analytical expression of the propagator in Eq (18) we start with the fact that $\xi^{⋆}=\alpha \sqrt{\delta t}\xi$ is Gaussian *N* ∼ (0, *δtα*^2^), hence the probability density function is known and must satisfy the following condition:

$$\begin{array}{c} \int_{-\infty }^{+\infty }\frac{1}{\sqrt{2\pi \delta t\alpha^{2}}}\exp\left(-\frac{{\xi^{⋆}}^{2}}{2\delta t\alpha^{2}}\right)d\xi^{⋆}=1. \end{array}$$ (16)

With an integration by substitution from Eq (9), with:

$$\begin{array}{c} \xi^{⋆}=\left(\left(\epsilon^{\prime }-\epsilon \right)\eta -\delta t\sigma_{0}+\delta tE\epsilon \right), \end{array}$$ (17)

we can then show the expression of the propagator, the probability that the system passes from state *ϵ* to *ϵ*′ during *δt* is given by [16]:

$$\begin{array}{c} W\left(\epsilon \to \epsilon^{\prime }\right)=\frac{\eta }{\sqrt{2\pi \delta t\alpha^{2}}}\exp\left(-\frac{{\left(\left(\epsilon^{\prime }-\epsilon \right)\eta -\delta t\sigma_{0}+\delta tE\epsilon \right)}^{2}}{2\delta t\alpha^{2}}\right). \end{array}$$ (18)

With the propagator in hand we will now see how it is possible to evaluate the Malliavin derivative with the MWS method. To recap, we denote by *J*(*ϵ*) a quantity of interest of our system and we want to compute the derivative of the mean value of this quantity of interest E[J] with respect to a parameter $\bar{m}$, in this case *σ*_0_ when *σ* = *σ*_0_ + *αξ*.

The form of the Malliavin weights *q*_*m*_ can be obtained using the following procedure. First, we know that with *dP*(*ϵ*) = *P*(*ϵ*)*dϵ*, Eq (1) we can write:

$$\begin{array}{c} E\left[J\right]=\int_{\epsilon }JP\left(\epsilon \right)d\epsilon , \end{array}$$ (19)

and by taking the derivative of Eq (19) [11, 16, 18]:

$$\begin{array}{c} \frac{\partial E[J]}{\partial \bar{m}}=\int_{\epsilon }JP\left(\epsilon \right)\frac{\partial \ln P}{\partial m}d\epsilon . \end{array}$$ (20)

To define a set of rules for updating *q*_*m*_, we differentiate Eq (14) with respect to *m*:

$$\begin{array}{c} P\left(\epsilon^{\prime }\right)\frac{\partial \ln P^{\prime }}{\partial \bar{m}}=\int_{\epsilon }W\left(\epsilon \to \epsilon^{\prime }\right)P\left(\epsilon \right)\frac{\partial \ln P+\partial \ln W}{\partial m}d\epsilon , \end{array}$$ (21)

and we obtain the following rule for updating the Malliavin weight:

$$\begin{array}{c} q_{m}\left(t+\delta t\right)=q_{m}\left(t\right)+\frac{\partial \ln W}{\partial m}. \end{array}$$ (22)

In the example of random stress with *σ* = *σ*_0_ + *ξ*, we obtain:

$$\begin{array}{c} \frac{\partial \ln W}{\partial \sigma }=\sqrt{\delta t}\xi /\alpha . \end{array}$$ (23)

For random Young’s modulus *E* = *E*_0_ + *ξ* we would have the same expression. In the case of random viscosity *η* = *η*_0_ + *ξ* we would obtain following the same logic:

$$\begin{array}{c} \frac{\partial \ln W}{\partial \eta }=\xi /\alpha . \end{array}$$ (24)

With the expression for the Malliavin weight Eq (23) in hand we can now implement an algorithm to calculate the derivative. The procedure is very simple; we take *Z* samples of the evolutions of the stochastic ODE using the explicit Euler scheme whilst simultaneously evolving the Malliavin weight *q*_*m*_. At teach time step Algorithm 1 describes this procedure in more detail.

The deterministic constants are given to be *η* = 1, *E* = 1 and we take a time step of *δt* = 0.01 for the finite difference scheme. We evaluate by the MWS method the derivative of the expected value of *ϵ* with respect to *σ*_0_ for a loading time *t* ∈ [0, *T*] with *T* = 30*s*. In this example the number of realisations is fixed at *Z* = 20000. We compare the results with the analytical solution which is:

$$\begin{array}{c} \frac{\partial E[\epsilon ]}{\partial \sigma_{0}}=\frac{1}{E}\left(1-\exp\left\{-\frac{Et}{\eta }\right\}\right). \end{array}$$ (25)

We briefly remark that for all numerical results presented in this paper there are two sources of errors committed with respect to the undiscretised problem. The first error is due to the deterministic approximation of the PDE (finite difference or finite element method), and the second due to the stochastic approximation (Monte Carlo estimator). In all cases we drive the error in the deterministic approximation of the PDE far lower than that in the stochastic approximation, such that the error is dominated by the number of realisations *Z* used in the Monte Carlo estimator.

**Fig 2:**
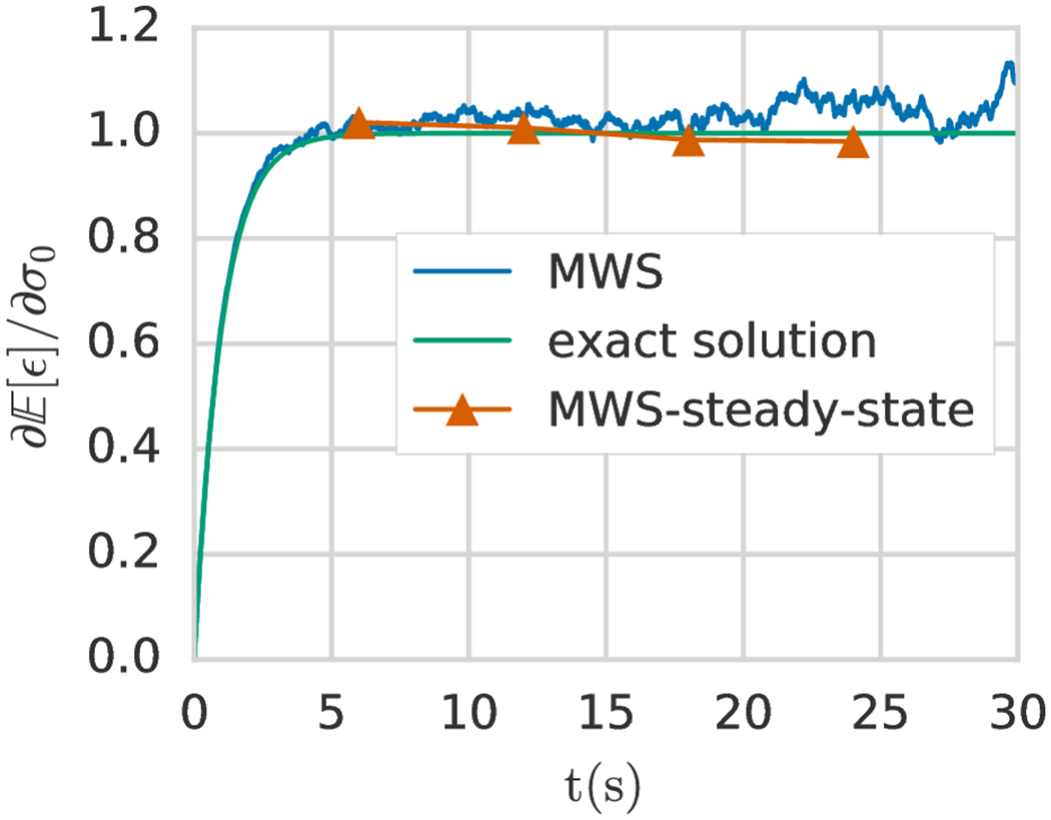
Malliavin derivative of the expected value of the strain with respect to the loading *σ*_0_ for the Kelvin-Voigt model with uncertain stress modelled as a Gaussian random variable. Comparison between the exact solution, the MWS method and the the MWS-steady-state method with a correction using the correlation function to improve the convergence of the MWS method when the system transitions into the steady state. For the MWS method we use *Z* = 20000 realisations at each time step.

In Fig 2 we can see that the MWS method gives a good estimation of the Malliavin derivative, particularly in the non-steady state regime *t* ≤ 5*s*. The relative error and so the global statistical error can become very high when the system reaches a steady state because the value of the sensitivity derivative is constant but the statistical error is compounded after each time step. To address this issue a technique can be employed based on the correlation function [16]. The reader is referred to [16] for further details. We have implemented this correlation correction, which we denote MWS-steady-state, and we can see in Fig 2 that the error is greatly reduced in the steady-state regime. For the numerical examples presented in the following sections, we will consider only the systems undergoing transition or purely steady state systems. Therefore we will not use the MWS-steady-state method again in this paper.

**Algorithm 1:** Malliavin Weight Sampling algorithm for time dependent problem. The notation used is that of the Kelvin-Voigt example in but the same basic algorithm is used throughout the paper. Note that a correction term is needed for systems in steady state, see [16].

**Data:** *σ*_0_, *E*, *η* and the random variable *ξ* ∈ *N*(0, 1).

**Result:** $\partial E\left[\epsilon \left(t\right)\right]/\partial \sigma_{0}$, the derivative of the mean of *ϵ* with respect to the mean stress *σ*_0_ at time *t*.

$\partial E\left[\epsilon \left(t\right)\right]/\partial \sigma_{0}=0$ for all t. ▹initialisation

**for** *z* = 0 to *Z* − 1 **do**

 *t* = 0; ▹time

 *ϵ*(*t*) = 0; ▹initial condition

 *q*_*σ*_ = 0; ▹MWS weight

 **for** *i* = 1 to *n* **do**

  Draw realisation of random variable *ξ*_*i*_;

$\epsilon \left(t+\delta t\right)=\epsilon \left(t\right)+\frac{\sigma_{0}\delta t}{\eta }-\frac{E\epsilon (t)\delta t}{\eta }+\frac{\sqrt{\delta t}\;\xi_{i}}{\eta }$;

$q_{\sigma }\left(t+\delta t\right)=q_{\sigma }\left(t\right)+\frac{\partial \ln W}{\partial \sigma }=q_{\sigma }+\sqrt{\delta t}\xi_{i}$;

$\partial E\left[\epsilon \left(t+\delta t\right)\right]/\partial \sigma_{0}+=\epsilon \left(t+\delta t\right)q_{\sigma }/Z$;

t+=δt;

 **end**

**end**

### Non-Gaussian case

In this section we explain how to calculate the derivative for non-Gaussian stochastic parameters using the MWS method. The procedure is similar to that shown in the previous part but the rule for updating the Malliavin weight must be modified.

We begin as before by considering uncertainty in the stress *σ*:

$$\begin{array}{ccc} \sigma & = & \sigma_{1}+c\xi \\ & = & \underset{\sigma_{0}}{\underset{︸}{\sigma_{1}+cE\left[\xi \right]}}+c\left(\xi -E\left[\xi \right]\right). \end{array}$$ (26)

where *ξ* a random variable with probability density function *f*(*x*) and *c* and *σ*_1_ are two constants. We have written Eq (26) in the form of a constant $\sigma_{0}=\sigma_{1}+cE\left[\zeta \right]$ plus a random variable c(ζ-E[ζ]) with zero mean. Then it follows that *σ*_0_ is the mean of the uncertain stress *σ*. We will use the MWS method to evaluate the derivative of the expected value of the quantity of interest with respect to *σ*_0_.

To be able to use the MWS method the probability density function on the parameter must satisfy some regularity conditions, see [11, 20, 21]. Intuitively, the probability density function must be sufficiently “regular” on R which holds for the Gaussian, log-normal, Beta(*α* > 1, *β* > 1) and Gamma(*k* > 1, *θ*) distributions. However, a uniform distribution between two values *a* and *b* can not be considered “regular” because the probability density function is not differentiable at *a* and *b*. Instead, we choose to regularised approximation of a uniform distribution using a Beta(1 + *e*, 1 + *e*) random variable with *e* ≪ 1.

Continuing, we again discretise Eq (5) using an explicit Euler method with time step *δt*:

$$\begin{array}{c} \epsilon \left(t+\delta t\right)=\epsilon \left(t\right)+\frac{\delta t}{\eta }\left(\sigma_{0}-E_{0}\epsilon \left(t\right)+\frac{c}{\sqrt{\delta t}}\left(\xi -E\left[\xi \right]\right)\right). \end{array}$$ (27)

Alternatively, in the case of uncertainty in the Young’s modulus, the discretisation can be written:

$$\begin{array}{c} \epsilon \left(t+\delta t\right)=\epsilon \left(t\right)+\frac{\delta t}{\eta }\left(\sigma_{0}-E_{0}\epsilon \left(t\right)+\frac{c\;\epsilon (t)}{\sqrt{\delta t}}\left(E\left[\xi \right]-\xi \right)\right), \end{array}$$ (28)

or, for uncertainty related to the viscosity:

$$\begin{array}{c} \epsilon \left(t+\delta t\right)=\epsilon \left(t\right)+\frac{\sigma_{0}-E\epsilon \left(t\right)}{\xi +\eta }\delta t. \end{array}$$ (29)

The probability density function of the beta distribution, for 0 ≤ *x* ≤ 1, and shape parameters *α*, *β* > 0, is:

$$\begin{array}{c} f\left(x;\alpha ,\beta \right)=\frac{1}{B(\alpha ,\beta )}x^{\alpha -1}{\left(1-x\right)}^{\beta -1}. \end{array}$$ (30)

The beta function *B* is a normalisation constant to ensure that the total probability integrates to 1. In general we will evaluate and update the Malliavin weight for the parameter *m* as:

$$\begin{array}{c} q_{m}\left(t+\delta t\right)=q_{m}\left(t\right)+\frac{\partial \ln W}{\partial m}=q_{m}\left(t\right)+\frac{\partial \ln f(\xi )}{\partial \xi }\frac{\partial \xi }{\partial m}. \end{array}$$ (31)

Note that in Eq (31), it is important to check that the condition $E[q_{m}\left(t\right)]=0$ is verified. If $E[q_{m}\left(t\right)]\neq 0$, the updated rule must be corrected. An example of performing this correction is given in the next section entitled extension to second derivative. Finally, we note that for the initial condition we always impose *q*_*m*_(*t* = 0) = 0.

For the uncertain Young’s modulus modelled with a beta distribution, we have:

$$\begin{array}{c} \frac{\partial \ln W}{\partial m}=\frac{\left(\beta -1\right)\sqrt{\delta t}}{c(1-m)}-\frac{\left(\alpha -1\right)\sqrt{\delta t}}{c\;m}. \end{array}$$ (32)

For the uncertain stress with beta distribution, we have:

$$\begin{array}{c} \frac{\partial \ln W}{\partial m}=\frac{\left(\beta -1\right)\sqrt{\delta t}}{c(1-m)}-\frac{\left(\alpha -1\right)\sqrt{\delta t}}{c\;m}. \end{array}$$ (33)

For the uncertain viscosity with beta distribution, we have:

$$\begin{array}{c} \frac{\partial \ln W}{\partial m}=\frac{\beta -1}{c(1-m)}-\frac{\alpha -1}{c\;m}. \end{array}$$ (34)

These results and further calculations are summarised in Table 1.

**Table 1:** Summary of main results for Kelvin-Voigt model with three distributions on three different model parameters.

distribution	$\frac{\partial \ln W}{\partial \sigma }$ or $\frac{\partial \ln W}{\partial E}$	$\frac{\partial \ln W}{\partial \eta }$
Beta(*α*, *β*)	$\frac{\left(\beta -1\right)\sqrt{\delta t}}{c(1-m)}-\frac{\left(\alpha -1\right)\sqrt{\delta t}}{mc}$	$\frac{\left(\beta -1\right)}{c(1-m)}-\frac{\left(\alpha -1\right)}{mc}$
Gamma(*κ*, *θ*)	$\frac{-\left(\kappa -1\right)\sqrt{\delta t}}{mc}+\frac{\sqrt{\delta t}}{\theta c}$	$\frac{-(\kappa -1)}{mc}+\frac{1}{\theta c}$
log-Normal(*μ*, *σ*)	$\frac{\sqrt{\delta t}}{mc}\left(1+\frac{\ln m-\mu }{\sigma^{2}}\right)$	$\frac{1}{mc}\left(1+\frac{\ln m-\mu }{\sigma^{2}}\right)$

**Fig 3:**
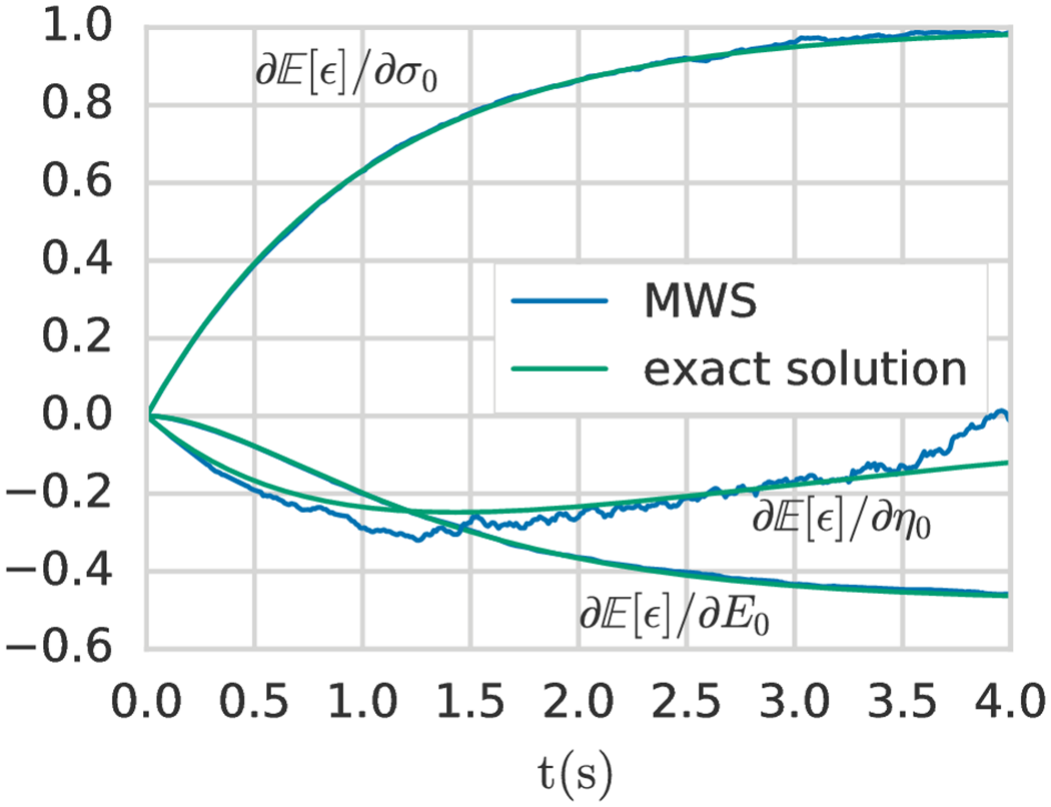
Malliavin derivatives of the expected value of the strain with respect to the mean of the stochastic parameters (Young’s modulus *E*_0_, viscosity *η*_0_ and stress loading *σ*_0_). Comparison between the exact solution and the MWS method. All uncertain parameters are modeled with a beta(2, 2) distribution. *Z* = 10^5^ realisations are performed for each estimator and the mean value of 10 estimators is plotted for each parameter. Note that the value of *Z* is not large enough for the viscosity to converge with an negligible error compared to the two other parameters. By increasing *Z*, this error could be reduced.

The Malliavin derivatives of the Kelvin-Voigt model with respect to the mean of the three parameters {*σ*_0_, *η*_0_, *E*_0_} modelled as beta(2, 2) distributions are shown in Fig 3. The exact solution is computed semi-analytically using standard integration rules. Good agreement between the MWS and semi-analytical solution is observed for *E*_0_ and *σ*_0_. For the viscosity *η*_0_ the number of Monte Carlo samples is not sufficient to achieve negligible error, but the overall trend is followed.

### Extension to second derivative

The MWS method can also be used to compute the second Malliavin derivative of the expected value of a quantity of interest *J*. If the quantity of interest does not depend explicitly of the random parameter, the expression given in Eq (3) is valid, but the more general form is the following:

$$\begin{array}{c} \frac{\partial E[J]}{\partial \bar{m}}=E\left[\frac{\partial J}{\partial \bar{m}}\right]+E\left[Jq_{m}\right]. \end{array}$$ (35)

In Eq (35), when we want to compute the second derivative the term $\left[\frac{\partial J}{\partial \bar{m}}\right]$ does not vanish because in this case *J* is the first derivative with respect to $\bar{m}$ and therefore depends on the parameter $\bar{m}$ in general. By applying Eq (35), we can show for example in the case of uncertain Young’s modulus that:

$$\begin{array}{c} \frac{\partial^{2}E\left[\epsilon \left(t\right)\right]}{\partial E_{0}^{2}}=E\left[\epsilon \left(t\right)\left(q_{EE}\left(t\right)+q_{E}{\left(t\right)}^{2}-C_{EE}-C_{E}^{2}\right)\right], \end{array}$$ (36)

with the following updating rule:

$$\begin{array}{c} q_{EE}\left(t+\delta t\right)=q_{EE}\left(t\right)+\frac{\partial^{2}\ln W}{\partial E^{2}}, \end{array}$$ (37)

and:

$$\begin{array}{c} q_{E}\left(t+\delta t\right)=q_{E}\left(t\right)+\frac{\partial \ln W}{\partial E}. \end{array}$$ (38)

The constant $C_{E}^{2}$ and *C*_*EE*_ allow to ensure that the expected value of the global Malliavin weight $\left(q_{EE}\left(t\right)+q_{E}{\left(t\right)}^{2}-C_{EE}-C_{E}^{2}\right)$ has an expected value equal to zero. In this specific case we have:

$$\begin{array}{c} C_{E}^{2}=E\left[{\left(\frac{\partial \ln W}{\partial E}\right)}^{2}\right], \end{array}$$ (39)

$$\begin{array}{c} C_{EE}=E\left[\frac{\partial^{2}\ln W}{\partial E^{2}}\right]. \end{array}$$ (40)

The precise specification of the constants depends on the distribution. We compute them analytically or by using standard numerical integration techniques found in e.g. Scipy or Maple.

**Fig 4:**
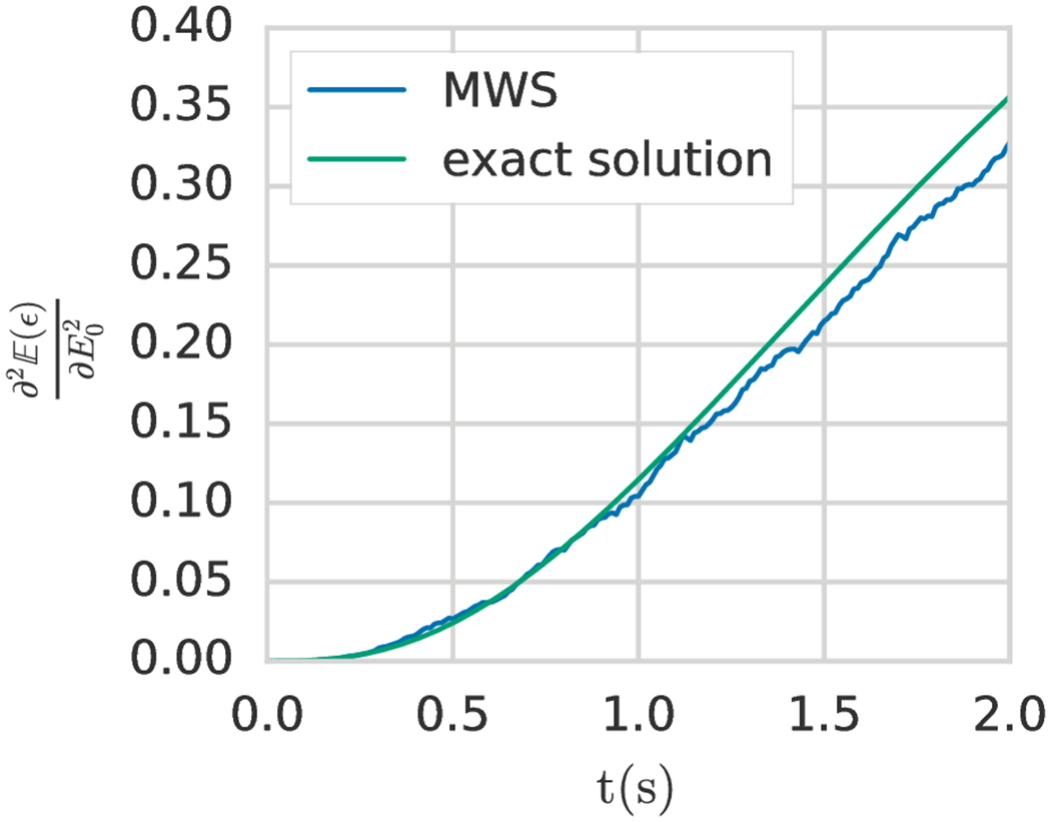
Second sensitivity derivative of the expected value of the strain with respect to the Young’s modulus for the Kelvin-Voigt model. Comparison between the exact solution solution and the MWS method. The Young’s modulus is modelled with a log-normal distribution. For the MWS method, *Z* = 10^7^ realisations are performed. Note that the value of *Z* for the same order of magnitude for the error is higher for the second derivative compared to the first derivative because the variance *V* in the Malliavin estimator is bigger and we know from the central limit theorem that the error is in $O(V^{1/2}Z^{-1/2})$.

In Fig 4, a comparison between the analytical solution and the MWS method is given for the value of the second derivative depending on time of the expected value of *ϵ* with respect to the Young’s modulus. For the sake of example, the problem specification is the same as in previous sections, with the exception that the random variable follows a log-normal(*μ*, *σ*) distribution with mean equal to 0.5 and standard deviation equal to 0.25 which corresponds to *μ* = −0.804 and *σ* = 0.473. The analytical solutions for the two constants *C*_*EE*_ and $C_{E}^{2}$ are in this case:

$$\begin{array}{c} C_{EE}=C_{E}^{2}=\left(1+\frac{1}{\sigma^{2}}\right)\exp\left(2\sigma^{2}-2\mu \right). \end{array}$$ (41)

As we can see in Fig 4, the MWS method gives a good approximation for the evaluation of the second derivative with *Z* = 10^7^ realisations.

### Extension to random process

In this paper we deal with random noise and in the next section we show numerical results of stochastic mechanics problems where models are defined as PDEs. The probability density function of the random variables used in these examples does not depend on time. Similarly to the Kelvin-Voigt model presented before, we study a time dependent problem in a finite dimensional space by splitting the time interval [0, *T*] into a finite number of increments. Note that it is also possible to take into account the random noise only at the initial time instead of generating random variables at each time step. It would be possible to extend this work to random process, e.g. by using a Wiener process *W*(*t*) which verifies in particular (*W*(*t* + *δt*) − *W*(*t*)) ∼ *N*(0, *δt*). In this case, even for simple ODEs, it is very difficult to obtain analytical solutions because the probability density function of a random process evolves in time. The Malliavin calculus is very well adapted to address these stochastic problems but requires much more advanced mathematical tools as those presented in this paper. In addition, the Malliavin calculus has the advantage and the specificity that it is possible to directly work in the continuum (infinite dimensional space) to evaluate the sensitivity derivatives. We hope that the first and simple approach restricted to random variables presented in this paper may be of interest to the engineering community and encourage them to investigate the benefits that the Malliavin calculus could provide in the context of stochastic PDEs.

## PDE examples

We now turn our attention to models that are defined as PDEs. To solve the deterministic evaluations of the PDEs we use the finite element method. We have chosen to use DOLFIN, part of the FEniCS Project to implement the finite element method solvers [22].

### Elastic bar with stochastic Young’s modulus

**Fig 5:**
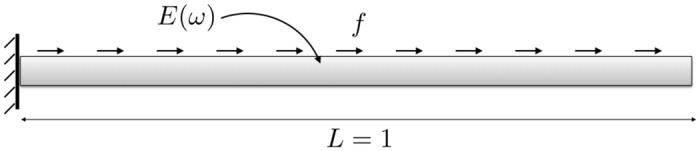
Elastic bar with stochastic Young’s modulus.

The strong form PDE and boundary conditions of the behaviour of a 1-dimensional elastic bar (see Fig 5) are:

$$\begin{array}{c} E\;\frac{d^{2}u\left(x\right)}{dx^{2}}+f=0;\;u\left(0\right)=0\;\text{and}\;\frac{du(L)}{dx}=0. \end{array}$$ (42)

We take *f* = 1, *L* = 1 and a stochastic Young’s modulus:

$$\begin{array}{c} E=2(1+\xi ), \end{array}$$ (43)

with *ξ* a random variable with beta(2, 2) distribution.

The forward model is described by the following weak residual formulation, find $u\in H_{D}^{1}\left(\Omega_{s}\right)$ such that:

$$\begin{array}{c} F\left(u;\tilde{u}\right)≔-\int_{\Omega_{s}}E\;\nabla u\cdot \nabla \tilde{u}\;dx+\int_{\Omega_{s}}f\tilde{u}\;dx=0\;\forall \tilde{u}\in H_{0}^{1}\left(\Omega_{s}\right), \end{array}$$ (44)

where the space $H_{D}^{1}\left(\Omega_{s}\right)$ is the usual Sobolev space of square-integrable functions with square-integrable weak derivatives on the domain Ω_*s*_ ≔ [0, 1] that satisfy the Dirichlet boundary condition *u*(0) = 0 and $H_{0}^{1}\left(\Omega_{s}\right)$ vanish on the whole boundary. We solve the forward model using a piecewise linear finite element method with 1024 cells in the mesh.

The quantity of interest is:

$$\begin{array}{c} J=\int_{0}^{1}u\left(x\right)dx. \end{array}$$ (45)

The derivative of the expected value of *J* with respect to the mean value of the Young’s modulus $\bar{E}$ can be computed analytically in this case:

$$\begin{array}{c} \frac{\partial E[J]}{\partial \bar{E}}=-\int_{0}^{1}\frac{1}{3{\left(2+2x\right)}^{2}}\frac{x(1-x)}{B(2,2)}\;dx=1-\frac{3}{2}\ln\left(2\right). \end{array}$$ (46)

This problem is a stationary (not time-dependent), in contrast to the Kelvin-Voigt model considered previously. However, this stationary problem can be solved using the same techniques. We introduce the concept of pseudo-time, where the system evolves from its initial state at *t* = 0 to the final solution at time *t* = *T* through the a single solution of the PDE Eq (44). Therefore in algorithm 1 we take the pseudo-time step as *δt* = *T* and hence *n* = 1. As before, the Malliavin weight still has initial condition *q*_*m*_(0) = 0.

Finally, the relative error between the MWS method with *Z* = 5 × 10^5^ realisations and the analytical solution is 3.0 × 10^−3^.

### Buckling of a hyperelastic beam with stochastic Young’s modulus

We study the deformation of a 2D geometrically non-linear hyperelastic beam with stochastic Young’s modulus *E*. We have deliberately designed this problem so that for some values of *E* the beam undergoes buckling, and for others not.

**Fig 6:**
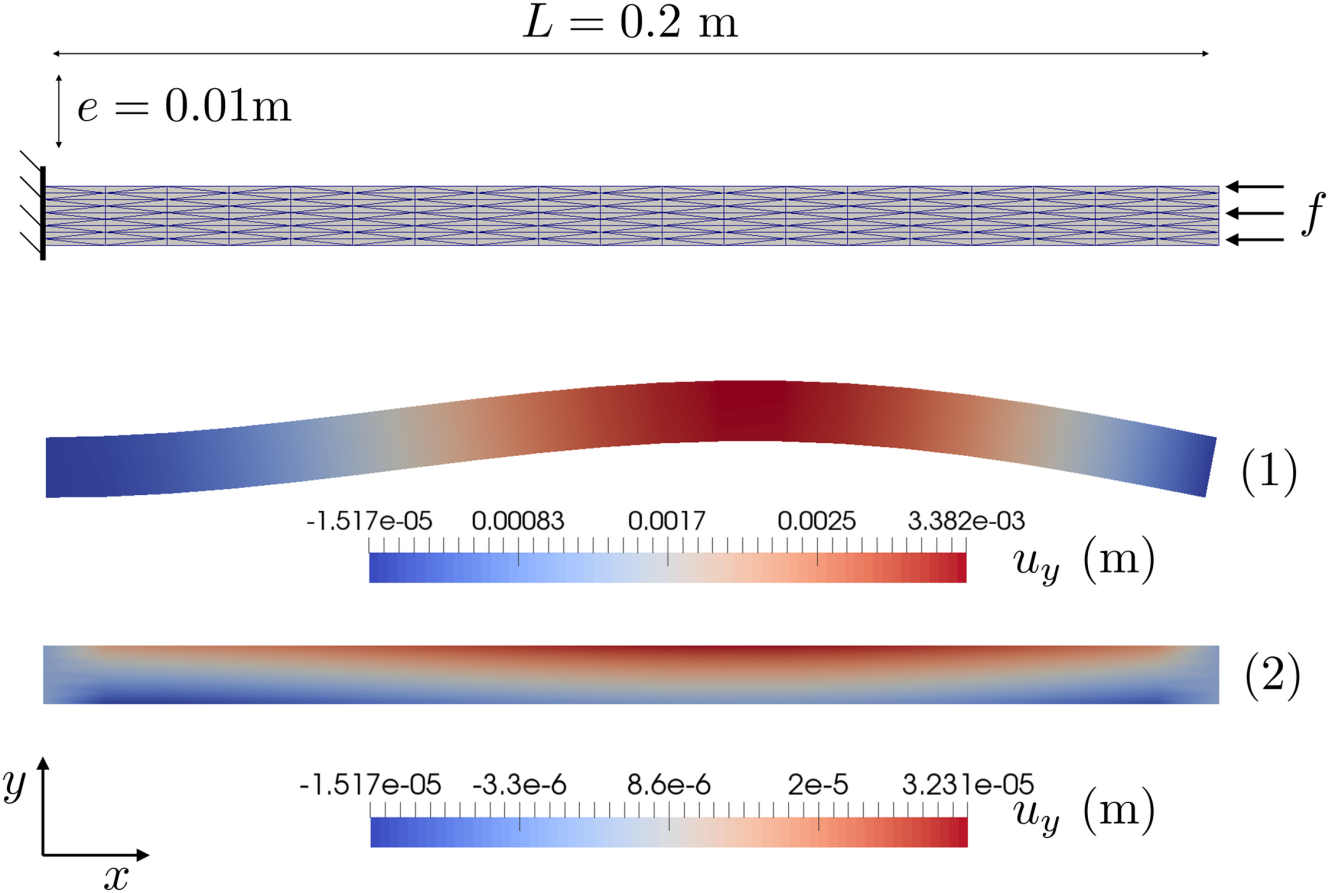
Hyperelastic beam: Mesh and schematic of boundary conditions. (1) a realisation of the problem where there is a geometric instability (buckling) and (2) another without.

Consider a hyperelastic body that in its undeformed state occupies the domain $\Omega_{0}=\left[0,L\right]\times \left[0,e\right]\subset R^{2}$ with *L* = 0.2*m* and *e* = 0.01*m* (see Fig 6), and in its deformed state occupies some (unknown) domain $\Omega \subset R^{2}$. ***φ*** is the map between the material points **X** in the undeformed domain Ω_0_ and points **x** in the deformed domain Ω:

$$\begin{array}{c} \varphi :\Omega_{0}∍X\to x\in \Omega , \end{array}$$ (47)

The deformation gradient can be written $F\left(X\right)≔\frac{\partial \varphi }{\partial X}$. The right Cauchy-Green tensor is then defined as $C≔F^{T}F$.

The Neo-Hookean stored energy density of the body is then:

$$\begin{array}{c} W\left(F\right)≔\mu \left(I_{c}-2\right)/2-\mu \log I_{3}+\lambda {\left(\log I_{3}\right)}^{2}/2. \end{array}$$ (48)

where $I_{3}≔\det\left(F\right)$ and and $I_{C}=\mathrm{tr}\left(C\right)$. *λ* and *μ* are the Lame parameters and can be expressed as a function of the Young’s modulus *E* and Poisson’s ratio *ν* as:

$$\begin{array}{c} \lambda =\frac{E\nu }{\left(1+\nu )(1-2\nu \right)}\;\text{and}\;\mu =\frac{E}{2(1+\nu )}. \end{array}$$ (49)

We choose to model the Young’s modulus as a log-normal random variable with mean value 11 *MPa* and standard deviation 2 *MPa*. We take Poisson’s ratio as a fixed constant *ν* = 0.3.

Defining the displacement field as **u** ≔ ***φ*** − **X** and a linear functional **f** that encodes the external tractions we can characterise the elastic equilibrium displacement field u* as the solution to the following minimisation problem:

$$\begin{array}{ccc} u^{*} & = & \underset{u\in {\left[H_{D}^{1}\left(\Omega_{0}\right)\right]}^{2}}{arg min}L\left(u\right)\\ & = & \underset{u\in {\left[H_{D}^{1}\left(\Omega_{0}\right)\right]}^{2}}{arg min}\left\{\int_{\Omega_{0}}W\left(F\right)\;dx_{0}-\left\langle f,u\right\rangle \right\}, \end{array}$$ (50)

where ${\left[H_{D}^{1}\left(\Omega_{0}\right)\right]}^{2}$ is the usual vector-valued Sobolev space of square integrable functions with square integrable derivatives that satisfies the given Dirichlet boundary conditions and *dx*_0_ is a measure on Ω_0_. We fix the left hand of the beam, *u*(0, *y*) = 0 and apply a surface traction in the negative *x* direction on the right hand of the beam of magnitude *f*.

For one Monte Carlo realisation we solve the non-linear problem using a Newton method from SNES [23] with continuation in the loading parameter *f* and a third-order backtracking line search. We let the symbolic differentiation capabilities of UFL derive the residual and Jacobian of the forward model for use in the Newton method. We solve the linear systems arising from the Newton iterations using a conjugate gradient method preconditioned using algebraic multigrid (Hypre BoomerAMG [24]) interfaced from PETSc [23].

The quantity of interest is defined as:

$$\begin{array}{c} J=\int_{\Omega }\left|u_{y}\right|\;dx. \end{array}$$ (51)

The Malliavin derivative of E[J] with respect to the mean Young’s modulus obtained with the MWS method with *Z* = 3 × 10^3^ realisations is:

$$\begin{array}{c} \frac{\partial E[J]}{\partial E_{0}}\approx -3.1\times 10^{-6}\;m^{3}/\text{MPa}. \end{array}$$ (52)

No analytical solution exists for comparison. If we use dolfin-adjoint [25], we can compute the classical derivative of *J* with respect to the Young’s modulus around the mean parameter:

$$\begin{array}{c} \frac{\partial J}{\partial E}{|}_{E=E_{0}}\approx -3.5\times 10^{-8}\;m^{3}/\text{MPa}. \end{array}$$ (53)

In this example the difference between the classical derivative and the Malliavin derivative is quite pronounced. This difference is caused by the presence of an instability (buckling). This instability is not activated when *E* = *E*_0_, hence, the classical derivative tells us that *J* is relatively insensitive to perturbations in the Young’s modulus about *E*_0_. However, the Malliavin derivative tells us that E[J] *is* in fact quite sensitive to changes in the mean of the Young’s modulus *E*_0_. The Malliavin derivative gives us quite a different perspective on the sensitivity of this problem than the classical one.

### Incompressible Navier-Stokes equations with stochastic viscosity

We consider the incompressible Navier-Stokes equations on a domain $\Omega \;\text{in}\;R^{2}$ consisting of a pair of momentum and continuity equations:

$$\begin{array}{ccc} \dot{u}+\nabla u\cdot u-\nu \Delta u+\nabla p & = & f,\\ \nabla \cdot u & = & 0. \end{array}$$ (54)

**Fig 7:**
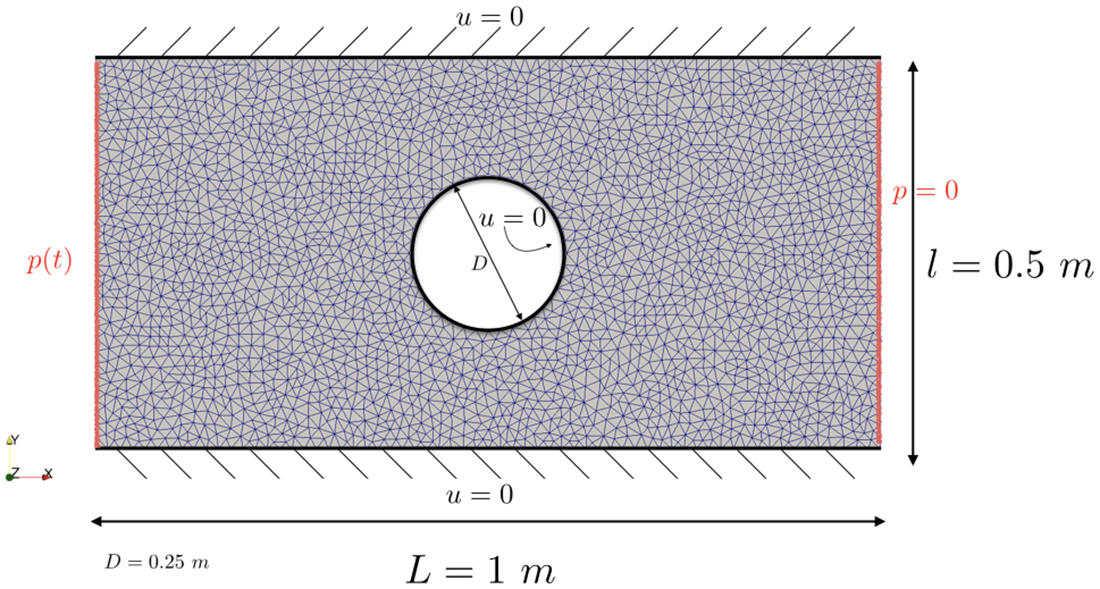
Mesh, geometry and boundary conditions for the incompressible Navier-Stokes problem.

In Eq (54), **u** refers to the unknown velocity of the fluid, *ν* is the viscosity of the fluid, *p* the unknown pressure and **f** is a given source. The mesh, geometry and boundary conditions for the incompressible Navier-Stokes problem are shown in Fig 7. The viscosity is modelled as a random variable:

$$\begin{array}{c} \nu =0.015+0.01(\xi -0.005), \end{array}$$ (55)

with *ξ* a log-normal distribution with mean equal to 0.5 and standard deviation equal to 0.25.

**Fig 8:**
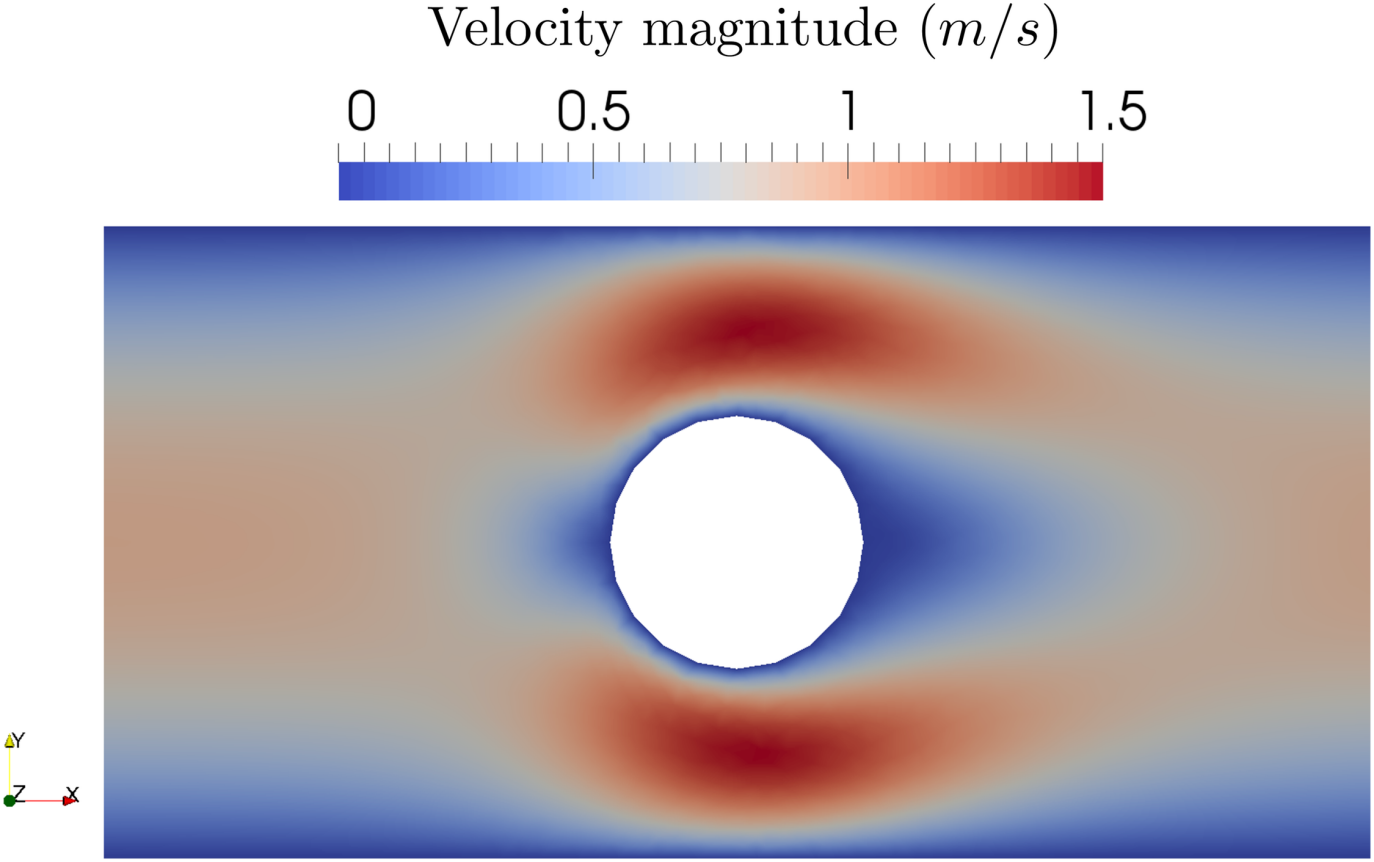
Velocity magnitude at time *t* = 1 s for one realisation of the viscosity.

We solve the PDE for a given parameter *ν* with FEniCS [26] (FE approximation) using Chorin’s method with time step *δt* = 0.01 for *t* ∈ [0, 1], see [27] for more details on the implementation. For one realisation of the viscosity the velocity at time *t* = 1 s is show in Fig 8.

The quantity of interest is the total volume of fluid that exits the right end of the domain:

$$\begin{array}{c} J=\int_{t=0}^{t=1}\int_{S_{p=0}}u\cdot n\;dsdt, \end{array}$$ (56)

where *S*_*p* = 0_ is the surface with normal vector **n** on the right side where the pressure is imposed to zero.

The derivative of E[J] with respect to *η*_0_ obtained with the MWS method for *Z* = 4 × 10^5^ realisations is:

$$\begin{array}{c} \frac{\partial E[J]}{\partial \eta_{0}}\approx -7.9\;s/m^{2}. \end{array}$$ (57)

No analytical solution exists for comparison. If we use dolfin-adjoint [25], we can compute the derivative of *J* with respect to the viscosity around the mean parameter:

$$\begin{array}{c} \frac{\partial J}{\partial \eta }{|}_{\eta =\eta_{0}}\approx -7.85\;s/m^{2}. \end{array}$$ (58)

The two sensitivity derivatives are close. In this example, contrary to the hyperelastic example, the Malliavin approach does not give us a particularly different interpretation of the sensitivity.

## Conclusion

In this paper we have shown how to calculate the Malliavin derivative using the method of Malliavin Weight Sampling. We have applied the method to some typical mechanics models that can be described by ODEs and PDEs, and solved those models using finite difference and finite element methods. In addition, we have extended the existing practical literature on MWS to non-Gaussian random variables and the calculation of second-order derivatives. We are currently investigating the extension of this work from random parameters to problems with variables modelled as random fields. We are also exploring the use of the Malliavin derivative in derivative-driven variance reduction methods e.g. [28]. Code showing the calculation of the Malliavin derivative for the examples in this paper is freely available at: https://dx.doi.org/10.6084/m9.figshare.5432722 [29].
